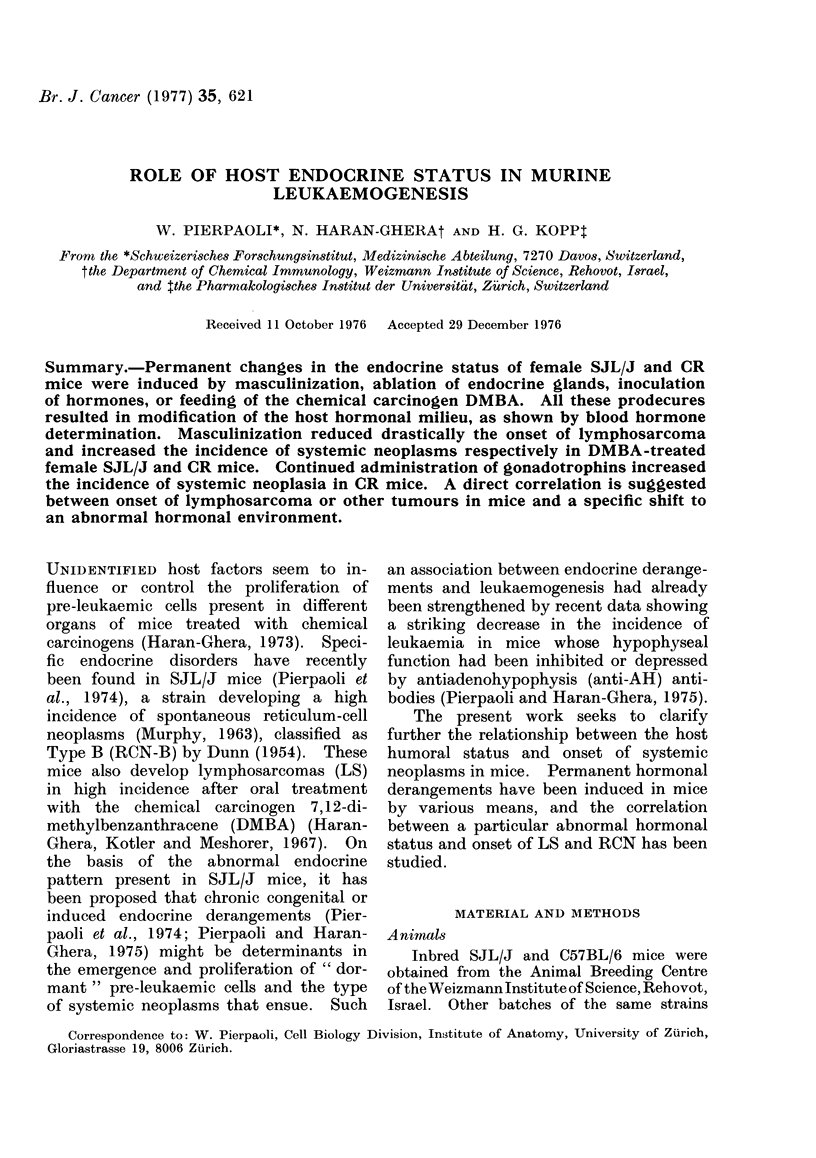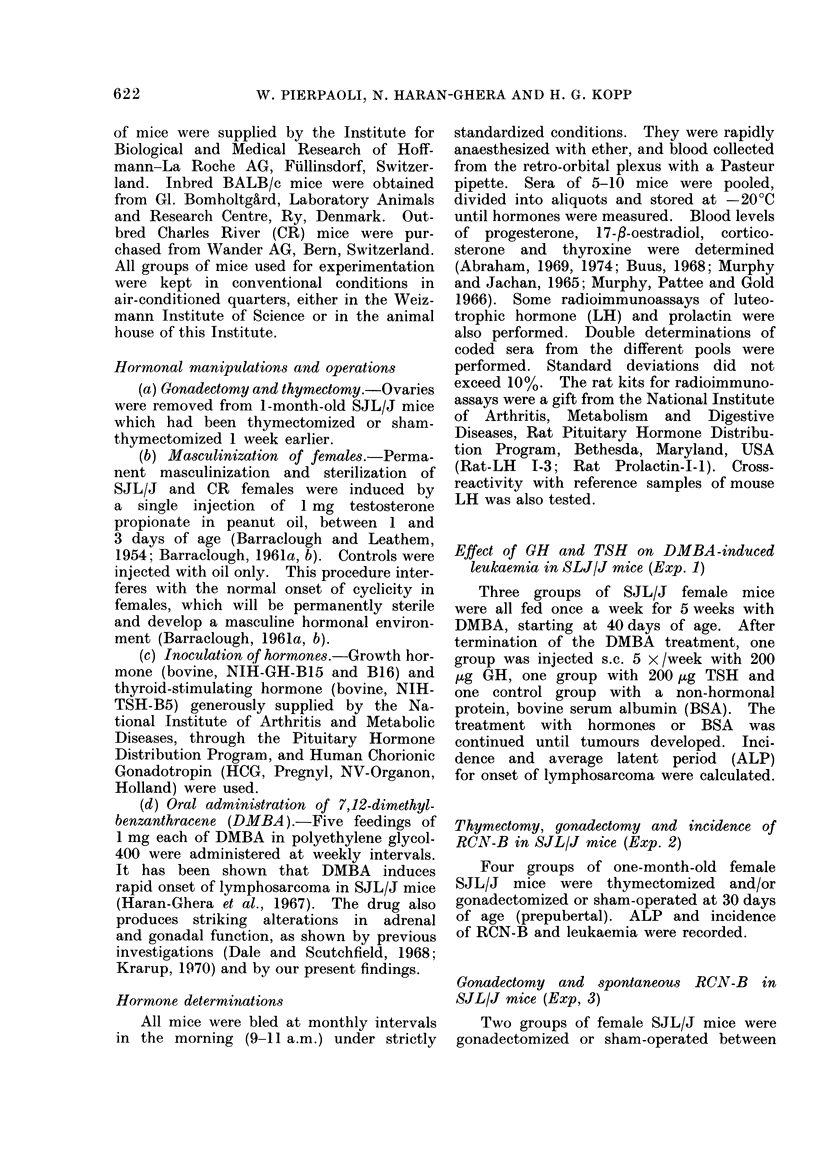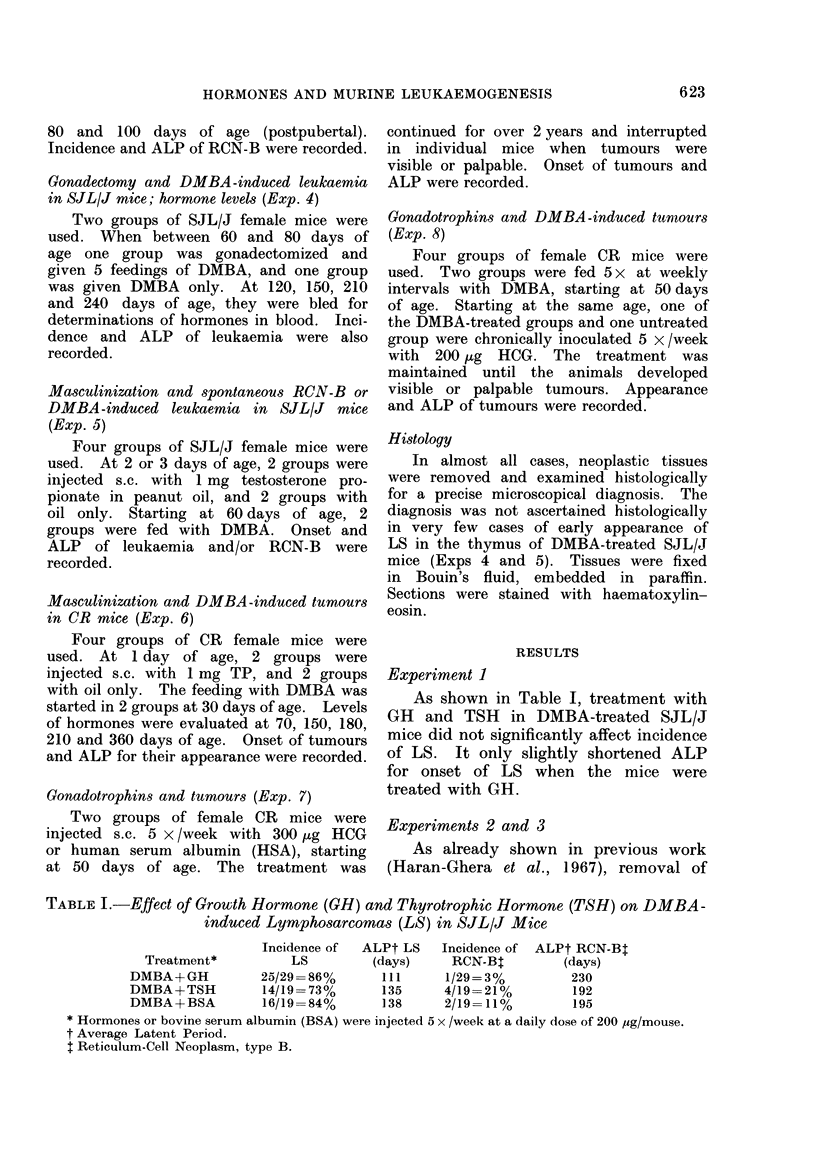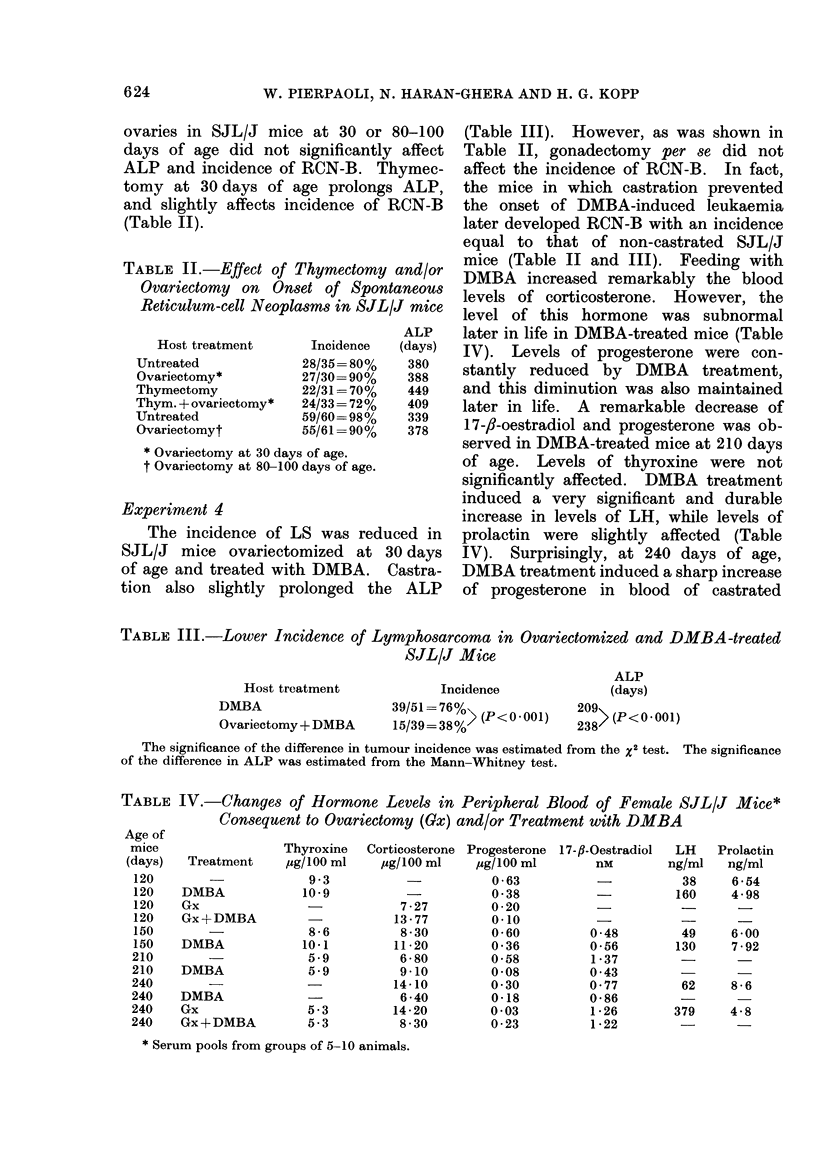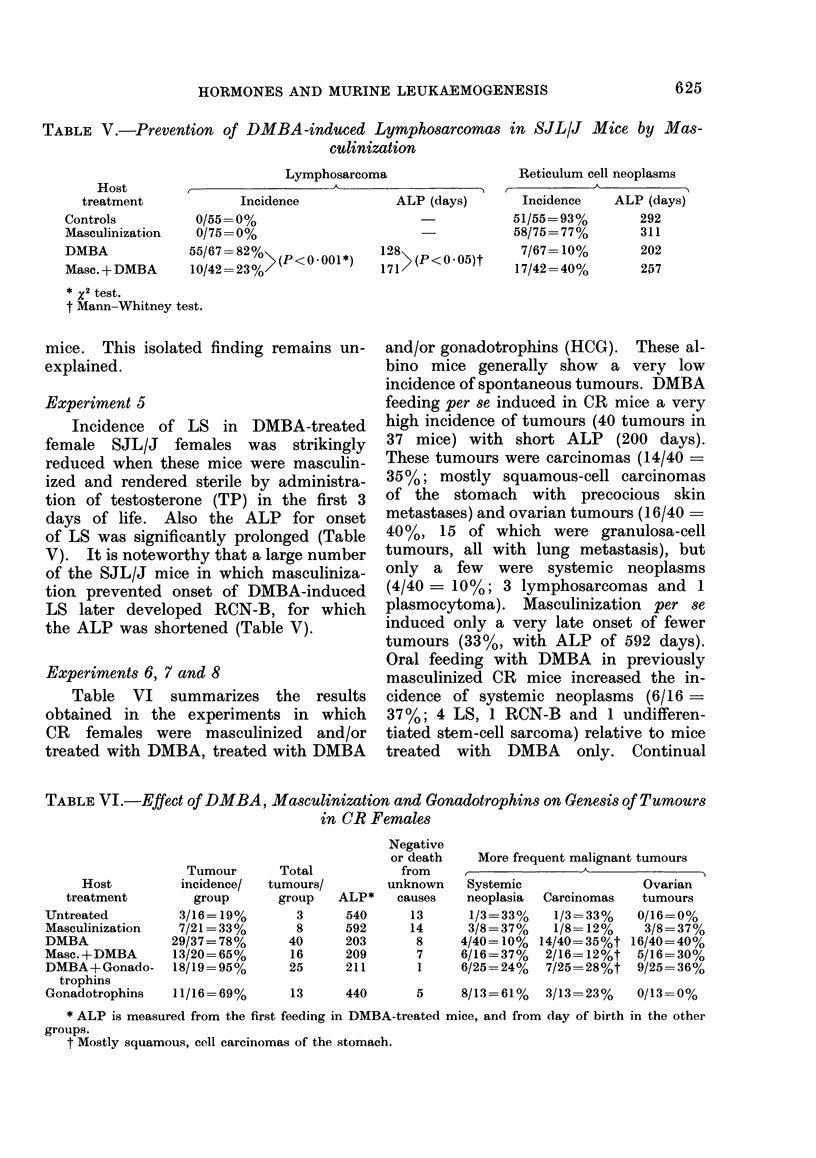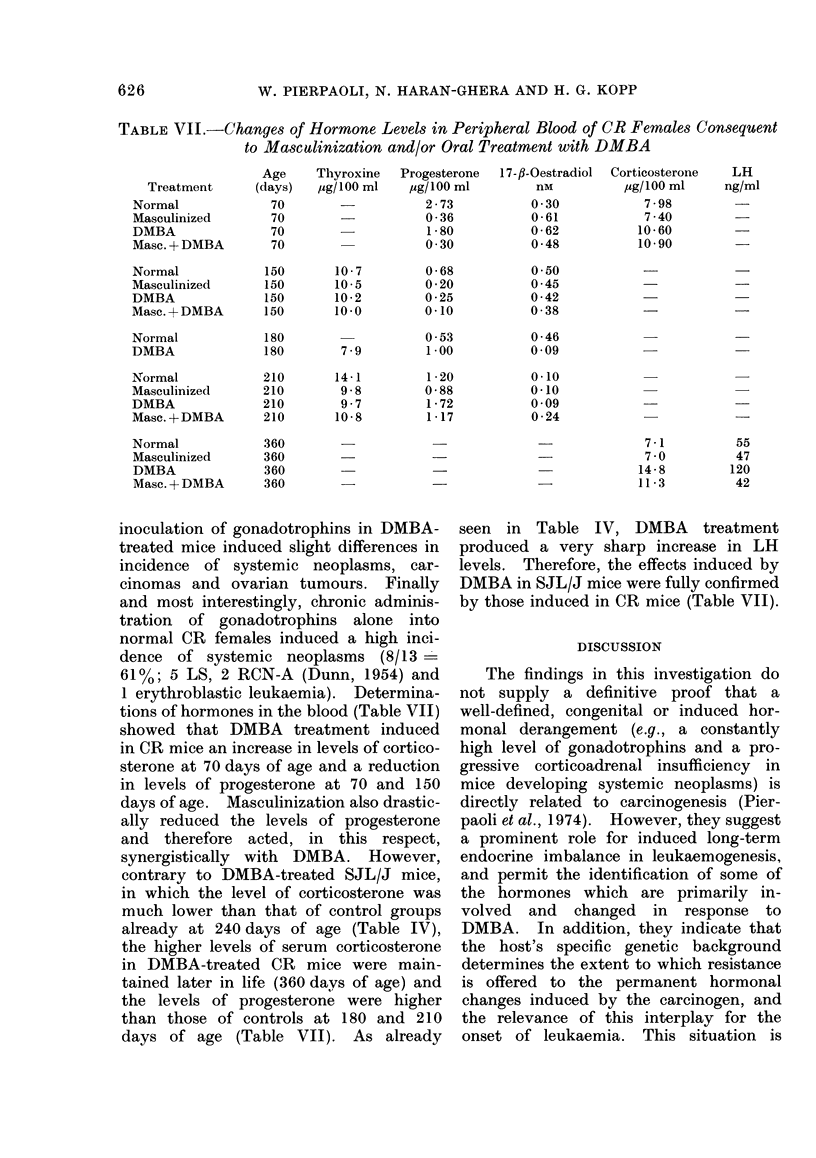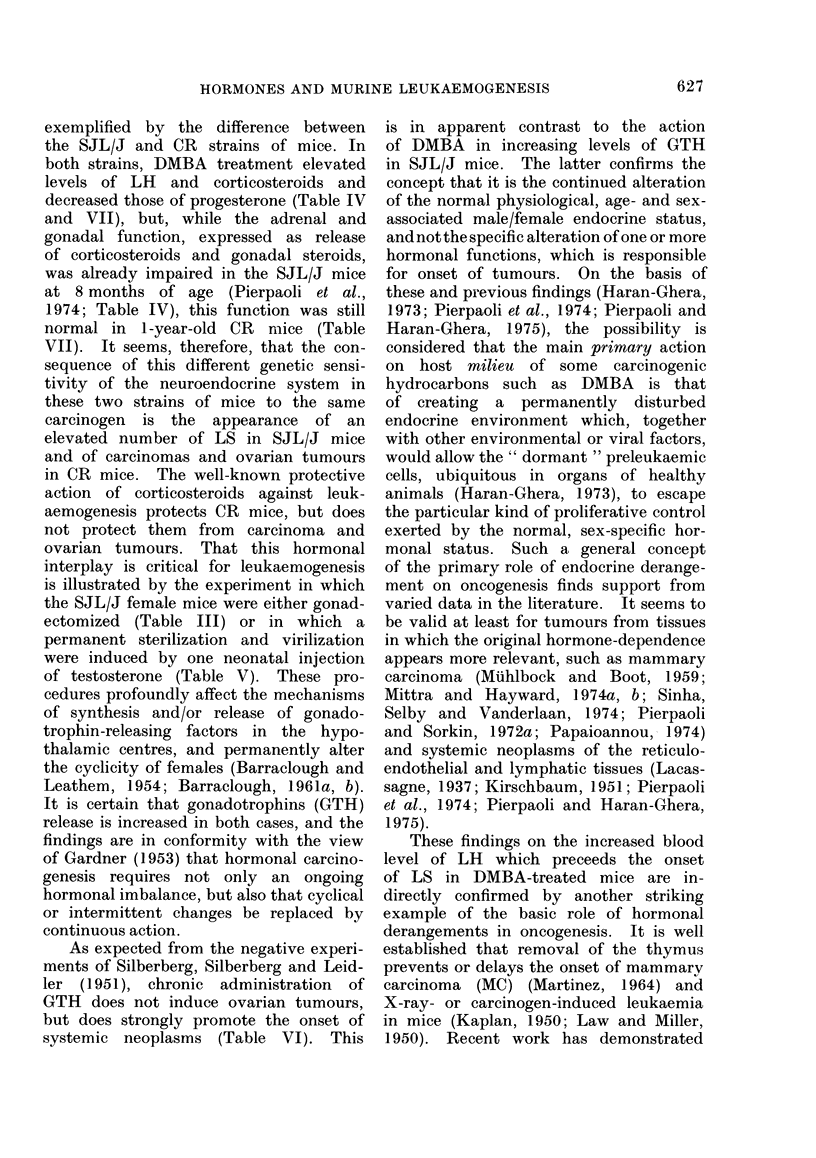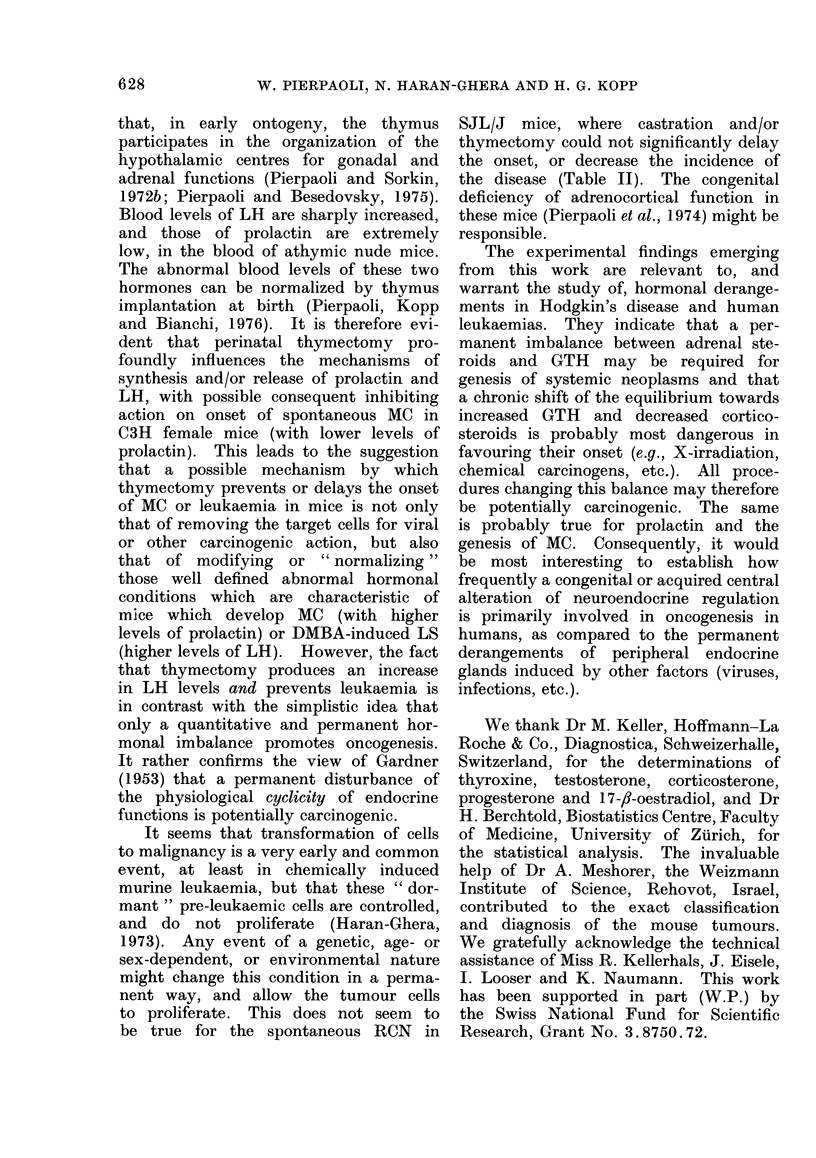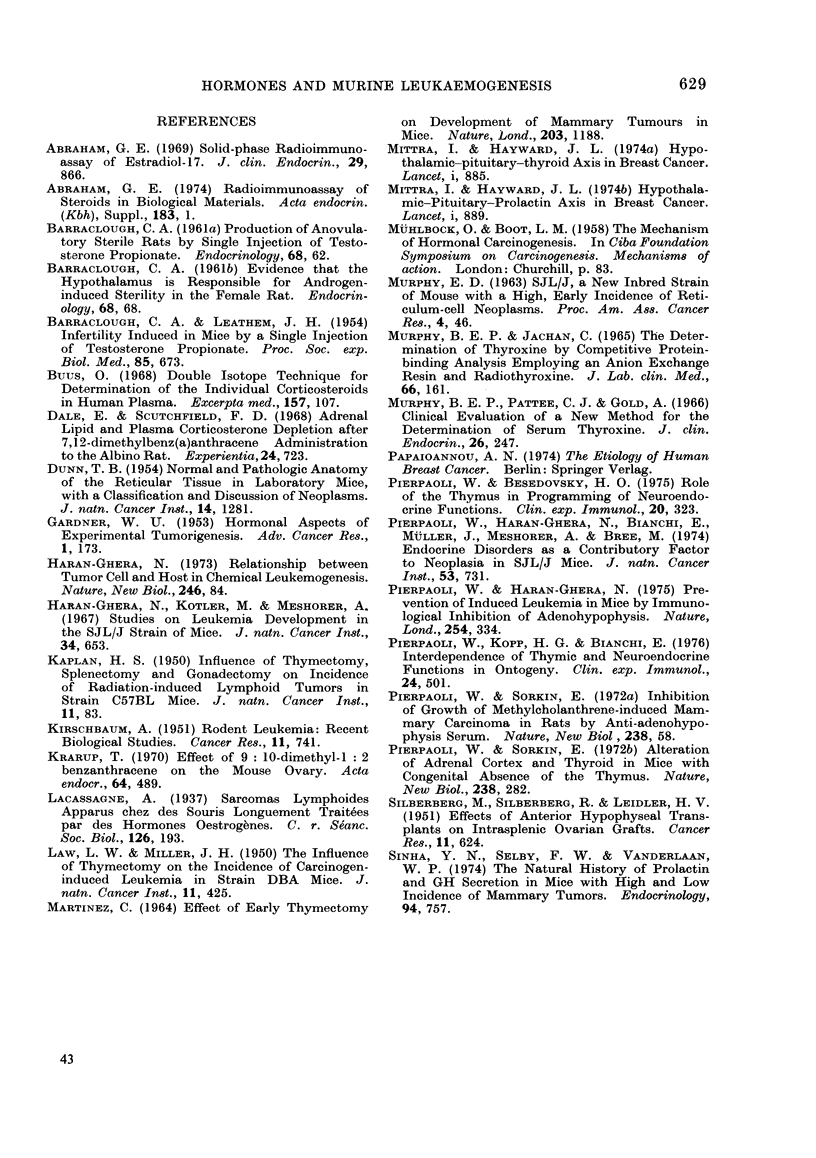# Role of host endocrine status in murine leukaemogenesis.

**DOI:** 10.1038/bjc.1977.97

**Published:** 1977-05

**Authors:** W. Pierpaoli, N. Haran-Ghera, H. G. Kopp

## Abstract

Permanent changes in the endocrine status of female SJL/J and CR mice were induced by masculinization, ablation of endocrine glands, inoculation of hormones, or feeding of the chemical carcinogen DMBA. All these procedures resulted in modification of the host hormonal milieu, as shown by blood hormone determination. Masculinization reduced drastically the onset of lymphosarcoma and increased the incidence of systemic neoplasms respectively in DMBA-treated female SJL/J and CR mice. Continued administration of gonadotrophins increased the incidence of systemic neoplasia in CR mice. A direct correlation is suggested between onset of lymphosarcoma or other tumours in mice and a specific shift to an abnormal hormonal environment.


*Br. J. Cancer* (1977) **35**, 621

### ROLE OF HOST ENDOCRINE STATUS IN MURINE LEUKAEMOGENESIS

W. PIERPAOLI*, N. HARAN-GHERA† AND H. G. KOPP‡

*From the *Schweizerisches Forschungsinstitut, Medizinische Abteilung, 7270 Davos, Switzerland, †the Department of Chemical Immunology, Weizmann Institute of Science, Rehovot, Israel, and ‡the Pharmakologisches Institut der Universität, Zürich, Switzerland*

Received 11 October 1976 Accepted 29 December 1976

**Summary.—Permanent changes in the endocrine status of female SJL/J and CR mice were induced by masculinization, ablation of endocrine glands, inoculation of hormones, or feeding of the chemical carcinogen DMBA. All these prodecures resulted in modification of the host hormonal milieu, as shown by blood hormone determination. Masculinization reduced drastically the onset of lymphosarcoma and increased the incidence of systemic neoplasms respectively in DMBA-treated female SJL/J and CR mice. Continued administration of gonadotrophins increased the incidence of systemic neoplasia in CR mice. A direct correlation is suggested between onset of lymphosarcoma or other tumours in mice and a specific shift to an abnormal hormonal environment.**

UNIDENTIFIED host factors seem to influence or control the proliferation of pre-leukaemic cells present in different organs of mice treated with chemical carcinogens (Haran-Ghera, 1973). Specific endocrine disorders have recently been found in SJL/J mice (Pierpaoli *et al.*, 1974), a strain developing a high incidence of spontaneous reticulum-cell neoplasms (Murphy, 1963), classified as Type B (RCN-B) by Dunn (1954). These mice also develop lymphosarcomas (LS) in high incidence after oral treatment with the chemical carcinogen 7,12-dimethylbenzanthracene (DMBA) (Haran-Ghera, Kotler and Meshorer, 1967). On the basis of the abnormal endocrine pattern present in SJL/J mice, it has been proposed that chronic congenital or induced endocrine derangements (Pierpaoli *et al.*, 1974; Pierpaoli and Haran-Ghera, 1975) might be determinants in the emergence and proliferation of " dormant " pre-leukaemic cells and the type of systemic neoplasms that ensue. Such an association between endocrine derangements and leukaemogenesis had already been strengthened by recent data showing a striking decrease in the incidence of leukaemia in mice whose hypophyseal function had been inhibited or depressed by antiadenohypophysis (anti-AH) antibodies (Pierpaoli and Haran-Ghera, 1975).

The present work seeks to clarify further the relationship between the host humoral status and onset of systemic neoplasms in mice. Permanent hormonal derangements have been induced in mice by various means, and the correlation between a particular abnormal hormonal status and onset of LS and RCN has been studied.

### MATERIAL AND METHODS

### *Animals*

Inbred SJL/J and C57BL/6 mice were obtained from the Animal Breeding Centre of the Weizmann Institute of Science, Rehovot, Israel. Other batches of the same strains

Correspondence to: W. Pierpaoli, Cell Biology Division, Institute of Anatomy, University of Zürich, Gloriastrasse 19, 8006 Zürich.

of mice were supplied by the Institute for Biological and Medical Research of Hoffmann–La Roche AG, Füllinsdorf, Switzerland. Inbred BALB/c mice were obtained from Gl. Bomholtgård, Laboratory Animals and Research Centre, Ry, Denmark. Outbred Charles River (CR) mice were purchased from Wander AG, Bern, Switzerland. All groups of mice used for experimentation were kept in conventional conditions in air-conditioned quarters, either in the Weizmann Institute of Science or in the animal house of this Institute.

*Hormonal manipulations and operations*

(*a*) *Gonadectomy and thymectomy.*—Ovaries were removed from 1-month-old SJL/J mice which had been thymectomized or sham-thymectomized 1 week earlier.

(*b*) *Masculinization of females.*—Permanent masculinization and sterilization of SJL/J and CR females were induced by a single injection of 1 mg testosterone propionate in peanut oil, between 1 and 3 days of age (Barraclough and Leathem, 1954; Barraclough, 1961*a*, *b*). Controls were injected with oil only. This procedure interferes with the normal onset of cyclicity in females, which will be permanently sterile and develop a masculine hormonal environment (Barraclough, 1961*a*, *b*).

(*c*) *Inoculation of hormones.*—Growth hormone (bovine, NIH-GH-B15 and B16) and thyroid-stimulating hormone (bovine, NIH-TSH-B5) generously supplied by the National Institute of Arthritis and Metabolic Diseases, through the Pituitary Hormone Distribution Program, and Human Chorionic Gonadotropin (HCG, Pregnyl, NV-Organon, Holland) were used.

(*d*) *Oral administration of 7,12-dimethylbenzanthracene* (*DMBA*).—Five feedings of 1 mg each of DMBA in polyethylene glycol-400 were administered at weekly intervals. It has been shown that DMBA induces rapid onset of lymphosarcoma in SJL/J mice (Haran-Ghera *et al.*, 1967). The drug also produces striking alterations in adrenal and gonadal function, as shown by previous investigations (Dale and Scutchfield, 1968; Krarup, 1970) and by our present findings.

*Hormone determinations*

All mice were bled at monthly intervals in the morning (9–11 a.m.) under strictly standardized conditions. They were rapidly anaesthesized with ether, and blood collected from the retro-orbital plexus with a Pasteur pipette. Sera of 5–10 mice were pooled, divided into aliquots and stored at −20°C until hormones were measured. Blood levels of progesterone, 17-$\beta$-oestradiol, corticosterone and thyroxine were determined (Abraham, 1969, 1974; Buus, 1968; Murphy and Jachan, 1965; Murphy, Pattee and Gold 1966). Some radioimmunoassays of luteotrophic hormone (LH) and prolactin were also performed. Double determinations of coded sera from the different pools were performed. Standard deviations did not exceed 10%. The rat kits for radioimmunoassays were a gift from the National Institute of Arthritis, Metabolism and Digestive Diseases, Rat Pituitary Hormone Distribution Program, Bethesda, Maryland, USA (Rat-LH I-3; Rat Prolactin-I-1). Cross-reactivity with reference samples of mouse LH was also tested.

*Effect of GH and TSH on DMBA-induced leukaemia in SLJ/J mice* (*Exp. 1*)

Three groups of SJL/J female mice were all fed once a week for 5 weeks with DMBA, starting at 40 days of age. After termination of the DMBA treatment, one group was injected s.c. 5 ×/week with 200 $\mu$g GH, one group with 200 $\mu$g TSH and one control group with a non-hormonal protein, bovine serum albumin (BSA). The treatment with hormones or BSA was continued until tumours developed. Incidence and average latent period (ALP) for onset of lymphosarcoma were calculated.

*Thymectomy, gonadectomy and incidence of RCN-B in SJL/J mice* (*Exp. 2*)

Four groups of one-month-old female SJL/J mice were thymectomized and/or gonadectomized or sham-operated at 30 days of age (prepubertal). ALP and incidence of RCN-B and leukaemia were recorded.

*Gonadectomy and spontaneous RCN-B in SJL/J mice* (*Exp, 3*)

Two groups of female SJL/J mice were gonadectomized or sham-operated between

80 and 100 days of age (postpubertal). Incidence and ALP of RCN-B were recorded.

*Gonadectomy and DMBA-induced leukaemia in SJL/J mice; hormone levels (Exp. 4)*

Two groups of SJL/J female mice were used. When between 60 and 80 days of age one group was gonadectomized and given 5 feedings of DMBA, and one group was given DMBA only. At 120, 150, 210 and 240 days of age, they were bled for determinations of hormones in blood. Incidence and ALP of leukaemia were also recorded.

*Masculinization and spontaneous RCN-B or DMBA-induced leukaemia in SJL/J mice (Exp. 5)*

Four groups of SJL/J female mice were used. At 2 or 3 days of age, 2 groups were injected s.c. with 1 mg testosterone propionate in peanut oil, and 2 groups with oil only. Starting at 60 days of age, 2 groups were fed with DMBA. Onset and ALP of leukaemia and/or RCN-B were recorded.

*Masculinization and DMBA-induced tumours in CR mice (Exp. 6)*

Four groups of CR female mice were used. At 1 day of age, 2 groups were injected s.c. with 1 mg TP, and 2 groups with oil only. The feeding with DMBA was started in 2 groups at 30 days of age. Levels of hormones were evaluated at 70, 150, 180, 210 and 360 days of age. Onset of tumours and ALP for their appearance were recorded.

*Gonadotrophins and tumours (Exp. 7)*

Two groups of female CR mice were injected s.c. 5 ×/week with 300 μg HCG or human serum albumin (HSA), starting at 50 days of age. The treatment was continued for over 2 years and interrupted in individual mice when tumours were visible or palpable. Onset of tumours and ALP were recorded.

*Gonadotrophins and DMBA-induced tumours (Exp. 8)*

Four groups of female CR mice were used. Two groups were fed 5× at weekly intervals with DMBA, starting at 50 days of age. Starting at the same age, one of the DMBA-treated groups and one untreated group were chronically inoculated 5 ×/week with 200 μg HCG. The treatment was maintained until the animals developed visible or palpable tumours. Appearance and ALP of tumours were recorded.

*Histology*

In almost all cases, neoplastic tissues were removed and examined histologically for a precise microscopical diagnosis. The diagnosis was not ascertained histologically in very few cases of early appearance of LS in the thymus of DMBA-treated SJL/J mice (Exps 4 and 5). Tissues were fixed in Bouin's fluid, embedded in paraffin. Sections were stained with haematoxylin-eosin.

### RESULTS

*Experiment 1*

As shown in Table I, treatment with GH and TSH in DMBA-treated SJL/J mice did not significantly affect incidence of LS. It only slightly shortened ALP for onset of LS when the mice were treated with GH.

*Experiments 2 and 3*

As already shown in previous work (Haran-Ghera *et al.*, 1967), removal of

TABLE I.—*Effect of Growth Hormone (GH) and Thyrotrophic Hormone (TSH) on DMBA-induced Lymphosarcomas (LS) in SJL/J Mice*

| Treatment* | Incidence of LS | ALP† LS (days) | Incidence of RCN-B‡ | ALP† RCN-B‡ (days) |
|---|---|---|---|---|
| DMBA+GH | 25/29=86% | 111 | 1/29=3% | 230 |
| DMBA+TSH | 14/19=73% | 135 | 4/19=21% | 192 |
| DMBA+BSA | 16/19=84% | 138 | 2/19=11% | 195 |

* Hormones or bovine serum albumin (BSA) were injected 5 ×/week at a daily dose of 200 μg/mouse.

† Average Latent Period.

‡ Reticulum-Cell Neoplasm, type B.

ovaries in SJL/J mice at 30 or 80–100 days of age did not significantly affect ALP and incidence of RCN-B. Thymectomy at 30 days of age prolongs ALP, and slightly affects incidence of RCN-B (Table II).

TABLE II.—*Effect of Thymectomy and/or Ovariectomy on Onset of Spontaneous Reticulum-cell Neoplasms in SJL/J mice*

| Host treatment | Incidence | ALP (days) |
|---|---|---|
| Untreated | 28/35 = 80% | 380 |
| Ovariectomy* | 27/30 = 90% | 388 |
| Thymectomy | 22/31 = 70% | 449 |
| Thym. + ovariectomy* | 24/33 = 72% | 409 |
| Untreated | 59/60 = 98% | 339 |
| Ovariectomy† | 55/61 = 90% | 378 |

* Ovariectomy at 30 days of age.
† Ovariectomy at 80–100 days of age.

*Experiment 4*

The incidence of LS was reduced in SJL/J mice ovariectomized at 30 days of age and treated with DMBA. Castration also slightly prolonged the ALP (Table III). However, as was shown in Table II, gonadectomy *per se* did not affect the incidence of RCN-B. In fact, the mice in which castration prevented the onset of DMBA-induced leukaemia later developed RCN-B with an incidence equal to that of non-castrated SJL/J mice (Table II and III). Feeding with DMBA increased remarkably the blood levels of corticosterone. However, the level of this hormone was subnormal later in life in DMBA-treated mice (Table IV). Levels of progesterone were constantly reduced by DMBA treatment, and this diminution was also maintained later in life. A remarkable decrease of 17-$\beta$-oestradiol and progesterone was observed in DMBA-treated mice at 210 days of age. Levels of thyroxine were not significantly affected. DMBA treatment induced a very significant and durable increase in levels of LH, while levels of prolactin were slightly affected (Table IV). Surprisingly, at 240 days of age, DMBA treatment induced a sharp increase of progesterone in blood of castrated

TABLE III.—*Lower Incidence of Lymphosarcoma in Ovariectomized and DMBA-treated SJL/J Mice*

| Host treatment | Incidence | | ALP (days) | |
|---|---|---|---|---|
| DMBA | 39/51 = 76% | ($P < 0.001$) | 209 | ($P < 0.001$) |
| Ovariectomy + DMBA | 15/39 = 38% | | 238 | |

The significance of the difference in tumour incidence was estimated from the $\chi^2$ test. The significance of the difference in ALP was estimated from the Mann–Whitney test.

TABLE IV.—*Changes of Hormone Levels in Peripheral Blood of Female SJL/J Mice* Consequent to Ovariectomy (Gx) and/or Treatment with DMBA*

| Age of mice (days) | Treatment | Thyroxine μg/100 ml | Corticosterone μg/100 ml | Progesterone μg/100 ml | 17-β-Oestradiol nM | LH ng/ml | Prolactin ng/ml |
|---|---|---|---|---|---|---|---|
| 120 | — | 9·3 | — | 0·63 | — | 38 | 6·54 |
| 120 | DMBA | 10·9 | — | 0·38 | — | 160 | 4·98 |
| 120 | Gx | — | 7·27 | 0·20 | — | — | — |
| 120 | Gx + DMBA | — | 13·77 | 0·10 | — | — | — |
| 150 | — | 8·6 | 8·30 | 0·60 | 0·48 | 49 | 6·00 |
| 150 | DMBA | 10·1 | 11·20 | 0·36 | 0·56 | 130 | 7·92 |
| 210 | — | 5·9 | 6·80 | 0·58 | 1·37 | — | — |
| 210 | DMBA | 5·9 | 9·10 | 0·08 | 0·43 | — | — |
| 240 | — | — | 14·10 | 0·30 | 0·77 | 62 | 8·6 |
| 240 | DMBA | — | 6·40 | 0·18 | 0·86 | — | — |
| 240 | Gx | 5·3 | 14·20 | 0·03 | 1·26 | 379 | 4·8 |
| 240 | Gx + DMBA | 5·3 | 8·30 | 0·23 | 1·22 | — | — |

* Serum pools from groups of 5–10 animals.

TABLE V.—*Prevention of DMBA-induced Lymphosarcomas in SJL/J Mice by Masculinization*

| Host treatment | Lymphosarcoma Incidence | | Lymphosarcoma ALP (days) | | Reticulum cell neoplasms Incidence | Reticulum cell neoplasms ALP (days) |
|---|---|---|---|---|---|---|
| Controls | 0/55 = 0% | | — | | 51/55 = 93% | 292 |
| Masculinization | 0/75 = 0% | | — | | 58/75 = 77% | 311 |
| DMBA | 55/67 = 82% | $(P<0\cdot001^*)$ | 128 | $(P<0\cdot05)\dagger$ | 7/67 = 10% | 202 |
| Masc. + DMBA | 10/42 = 23% | | 171 | | 17/42 = 40% | 257 |

* $\chi^2$ test.

† Mann–Whitney test.

mice. This isolated finding remains unexplained.

*Experiment 5*

Incidence of LS in DMBA-treated female SJL/J females was strikingly reduced when these mice were masculinized and rendered sterile by administration of testosterone (TP) in the first 3 days of life. Also the ALP for onset of LS was significantly prolonged (Table V). It is noteworthy that a large number of the SJL/J mice in which masculinization prevented onset of DMBA-induced LS later developed RCN-B, for which the ALP was shortened (Table V).

*Experiments 6, 7 and 8*

Table VI summarizes the results obtained in the experiments in which CR females were masculinized and/or treated with DMBA, treated with DMBA and/or gonadotrophins (HCG). These albino mice generally show a very low incidence of spontaneous tumours. DMBA feeding *per se* induced in CR mice a very high incidence of tumours (40 tumours in 37 mice) with short ALP (200 days). These tumours were carcinomas (14/40 = 35%; mostly squamous-cell carcinomas of the stomach with precocious skin metastases) and ovarian tumours (16/40 = 40%, 15 of which were granulosa-cell tumours, all with lung metastasis), but only a few were systemic neoplasms (4/40 = 10%; 3 lymphosarcomas and 1 plasmocytoma). Masculinization *per se* induced only a very late onset of fewer tumours (33%, with ALP of 592 days). Oral feeding with DMBA in previously masculinized CR mice increased the incidence of systemic neoplasms (6/16 = 37%; 4 LS, 1 RCN-B and 1 undifferentiated stem-cell sarcoma) relative to mice treated with DMBA only. Continual

TABLE VI.—*Effect of DMBA, Masculinization and Gonadotrophins on Genesis of Tumours in CR Females*

| Host treatment | Tumour incidence/ group | Total tumours/ group | ALP* | Negative or death from unknown causes | More frequent malignant tumours: Systemic neoplasia | Carcinomas | Ovarian tumours |
|---|---|---|---|---|---|---|---|
| Untreated | 3/16 = 19% | 3 | 540 | 13 | 1/3 = 33% | 1/3 = 33% | 0/16 = 0% |
| Masculinization | 7/21 = 33% | 8 | 592 | 14 | 3/8 = 37% | 1/8 = 12% | 3/8 = 37% |
| DMBA | 29/37 = 78% | 40 | 203 | 8 | 4/40 = 10% | 14/40 = 35%† | 16/40 = 40% |
| Masc. + DMBA | 13/20 = 65% | 16 | 209 | 7 | 6/16 = 37% | 2/16 = 12%† | 5/16 = 30% |
| DMBA + Gonadotrophins | 18/19 = 95% | 25 | 211 | 1 | 6/25 = 24% | 7/25 = 28%† | 9/25 = 36% |
| Gonadotrophins | 11/16 = 69% | 13 | 440 | 5 | 8/13 = 61% | 3/13 = 23% | 0/13 = 0% |

* ALP is measured from the first feeding in DMBA-treated mice, and from day of birth in the other groups.
† Mostly squamous, cell carcinomas of the stomach.

TABLE VII.—*Changes of Hormone Levels in Peripheral Blood of CR Females Consequent to Masculinization and/or Oral Treatment with DMBA*

| Treatment | Age (days) | Thyroxine μg/100 ml | Progesterone μg/100 ml | 17-β-Oestradiol nM | Corticosterone μg/100 ml | LH ng/ml |
|---|---|---|---|---|---|---|
| Normal | 70 | — | 2·73 | 0·30 | 7·98 | — |
| Masculinized | 70 | — | 0·36 | 0·61 | 7·40 | — |
| DMBA | 70 | — | 1·80 | 0·62 | 10·60 | — |
| Masc.+DMBA | 70 | — | 0·30 | 0·48 | 10·90 | — |
| Normal | 150 | 10·7 | 0·68 | 0·50 | — | — |
| Masculinized | 150 | 10·5 | 0·20 | 0·45 | — | — |
| DMBA | 150 | 10·2 | 0·25 | 0·42 | — | — |
| Masc.+DMBA | 150 | 10·0 | 0·10 | 0·38 | — | — |
| Normal | 180 | — | 0·53 | 0·46 | — | — |
| DMBA | 180 | 7·9 | 1·00 | 0·09 | — | — |
| Normal | 210 | 14·1 | 1·20 | 0·10 | — | — |
| Masculinized | 210 | 9·8 | 0·88 | 0·10 | — | — |
| DMBA | 210 | 9·7 | 1·72 | 0·09 | — | — |
| Masc.+DMBA | 210 | 10·8 | 1·17 | 0·24 | — | — |
| Normal | 360 | — | — | — | 7·1 | 55 |
| Masculinized | 360 | — | — | — | 7·0 | 47 |
| DMBA | 360 | — | — | — | 14·8 | 120 |
| Masc.+DMBA | 360 | — | — | — | 11·3 | 42 |

inoculation of gonadotrophins in DMBA-treated mice induced slight differences in incidence of systemic neoplasms, carcinomas and ovarian tumours. Finally and most interestingly, chronic administration of gonadotrophins alone into normal CR females induced a high incidence of systemic neoplasms (8/13 = 61%; 5 LS, 2 RCN-A (Dunn, 1954) and 1 erythroblastic leukaemia). Determinations of hormones in the blood (Table VII) showed that DMBA treatment induced in CR mice an increase in levels of corticosterone at 70 days of age and a reduction in levels of progesterone at 70 and 150 days of age. Masculinization also drastically reduced the levels of progesterone and therefore acted, in this respect, synergistically with DMBA. However, contrary to DMBA-treated SJL/J mice, in which the level of corticosterone was much lower than that of control groups already at 240 days of age (Table IV), the higher levels of serum corticosterone in DMBA-treated CR mice were maintained later in life (360 days of age) and the levels of progesterone were higher than those of controls at 180 and 210 days of age (Table VII). As already seen in Table IV, DMBA treatment produced a very sharp increase in LH levels. Therefore, the effects induced by DMBA in SJL/J mice were fully confirmed by those induced in CR mice (Table VII).

### DISCUSSION

The findings in this investigation do not supply a definitive proof that a well-defined, congenital or induced hormonal derangement (*e.g.*, a constantly high level of gonadotrophins and a progressive corticoadrenal insufficiency in mice developing systemic neoplasms) is directly related to carcinogenesis (Pierpaoli *et al.*, 1974). However, they suggest a prominent role for induced long-term endocrine imbalance in leukaemogenesis, and permit the identification of some of the hormones which are primarily involved and changed in response to DMBA. In addition, they indicate that the host's specific genetic background determines the extent to which resistance is offered to the permanent hormonal changes induced by the carcinogen, and the relevance of this interplay for the onset of leukaemia. This situation is exemplified by the difference between the SJL/J and CR strains of mice. In both strains, DMBA treatment elevated levels of LH and corticosteroids and decreased those of progesterone (Table IV and VII), but, while the adrenal and gonadal function, expressed as release of corticosteroids and gonadal steroids, was already impaired in the SJL/J mice at 8 months of age (Pierpaoli *et al.*, 1974; Table IV), this function was still normal in 1-year-old CR mice (Table VII). It seems, therefore, that the consequence of this different genetic sensitivity of the neuroendocrine system in these two strains of mice to the same carcinogen is the appearance of an elevated number of LS in SJL/J mice and of carcinomas and ovarian tumours in CR mice. The well-known protective action of corticosteroids against leukaemogenesis protects CR mice, but does not protect them from carcinoma and ovarian tumours. That this hormonal interplay is critical for leukaemogenesis is illustrated by the experiment in which the SJL/J female mice were either gonadectomized (Table III) or in which a permanent sterilization and virilization were induced by one neonatal injection of testosterone (Table V). These procedures profoundly affect the mechanisms of synthesis and/or release of gonadotrophin-releasing factors in the hypothalamic centres, and permanently alter the cyclicity of females (Barraclough and Leathem, 1954; Barraclough, 1961*a*, *b*). It is certain that gonadotrophins (GTH) release is increased in both cases, and the findings are in conformity with the view of Gardner (1953) that hormonal carcinogenesis requires not only an ongoing hormonal imbalance, but also that cyclical or intermittent changes be replaced by continuous action.

As expected from the negative experiments of Silberberg, Silberberg and Leidler (1951), chronic administration of GTH does not induce ovarian tumours, but does strongly promote the onset of systemic neoplasms (Table VI). This is in apparent contrast to the action of DMBA in increasing levels of GTH in SJL/J mice. The latter confirms the concept that it is the continued alteration of the normal physiological, age- and sex-associated male/female endocrine status, and not the specific alteration of one or more hormonal functions, which is responsible for onset of tumours. On the basis of these and previous findings (Haran-Ghera, 1973; Pierpaoli *et al.*, 1974; Pierpaoli and Haran-Ghera, 1975), the possibility is considered that the main *primary* action on host *milieu* of some carcinogenic hydrocarbons such as DMBA is that of creating a permanently disturbed endocrine environment which, together with other environmental or viral factors, would allow the " dormant " preleukaemic cells, ubiquitous in organs of healthy animals (Haran-Ghera, 1973), to escape the particular kind of proliferative control exerted by the normal, sex-specific hormonal status. Such a general concept of the primary role of endocrine derangement on oncogenesis finds support from varied data in the literature. It seems to be valid at least for tumours from tissues in which the original hormone-dependence appears more relevant, such as mammary carcinoma (Mühlbock and Boot, 1959; Mittra and Hayward, 1974*a*, *b*; Sinha, Selby and Vanderlaan, 1974; Pierpaoli and Sorkin, 1972*a*; Papaioannou, 1974) and systemic neoplasms of the reticuloendothelial and lymphatic tissues (Lacassagne, 1937; Kirschbaum, 1951; Pierpaoli *et al.*, 1974; Pierpaoli and Haran-Ghera, 1975).

These findings on the increased blood level of LH which preceeds the onset of LS in DMBA-treated mice are indirectly confirmed by another striking example of the basic role of hormonal derangements in oncogenesis. It is well established that removal of the thymus prevents or delays the onset of mammary carcinoma (MC) (Martinez, 1964) and X-ray- or carcinogen-induced leukaemia in mice (Kaplan, 1950; Law and Miller, 1950). Recent work has demonstrated that, in early ontogeny, the thymus participates in the organization of the hypothalamic centres for gonadal and adrenal functions (Pierpaoli and Sorkin, 1972*b*; Pierpaoli and Besedovsky, 1975). Blood levels of LH are sharply increased, and those of prolactin are extremely low, in the blood of athymic nude mice. The abnormal blood levels of these two hormones can be normalized by thymus implantation at birth (Pierpaoli, Kopp and Bianchi, 1976). It is therefore evident that perinatal thymectomy profoundly influences the mechanisms of synthesis and/or release of prolactin and LH, with possible consequent inhibiting action on onset of spontaneous MC in C3H female mice (with lower levels of prolactin). This leads to the suggestion that a possible mechanism by which thymectomy prevents or delays the onset of MC or leukaemia in mice is not only that of removing the target cells for viral or other carcinogenic action, but also that of modifying or "normalizing" those well defined abnormal hormonal conditions which are characteristic of mice which develop MC (with higher levels of prolactin) or DMBA-induced LS (higher levels of LH). However, the fact that thymectomy produces an increase in LH levels *and* prevents leukaemia is in contrast with the simplistic idea that only a quantitative and permanent hormonal imbalance promotes oncogenesis. It rather confirms the view of Gardner (1953) that a permanent disturbance of the physiological *cyclicity* of endocrine functions is potentially carcinogenic.

It seems that transformation of cells to malignancy is a very early and common event, at least in chemically induced murine leukaemia, but that these "dormant" pre-leukaemic cells are controlled, and do not proliferate (Haran-Ghera, 1973). Any event of a genetic, age- or sex-dependent, or environmental nature might change this condition in a permanent way, and allow the tumour cells to proliferate. This does not seem to be true for the spontaneous RCN in SJL/J mice, where castration and/or thymectomy could not significantly delay the onset, or decrease the incidence of the disease (Table II). The congenital deficiency of adrenocortical function in these mice (Pierpaoli *et al.*, 1974) might be responsible.

The experimental findings emerging from this work are relevant to, and warrant the study of, hormonal derangements in Hodgkin's disease and human leukaemias. They indicate that a permanent imbalance between adrenal steroids and GTH may be required for genesis of systemic neoplasms and that a chronic shift of the equilibrium towards increased GTH and decreased corticosteroids is probably most dangerous in favouring their onset (*e.g.*, X-irradiation, chemical carcinogens, etc.). All procedures changing this balance may therefore be potentially carcinogenic. The same is probably true for prolactin and the genesis of MC. Consequently, it would be most interesting to establish how frequently a congenital or acquired central alteration of neuroendocrine regulation is primarily involved in oncogenesis in humans, as compared to the permanent derangements of peripheral endocrine glands induced by other factors (viruses, infections, etc.).

We thank Dr M. Keller, Hoffmann–La Roche & Co., Diagnostica, Schweizerhalle, Switzerland, for the determinations of thyroxine, testosterone, corticosterone, progesterone and 17-$\beta$-oestradiol, and Dr H. Berchtold, Biostatistics Centre, Faculty of Medicine, University of Zürich, for the statistical analysis. The invaluable help of Dr A. Meshorer, the Weizmann Institute of Science, Rehovot, Israel, contributed to the exact classification and diagnosis of the mouse tumours. We gratefully acknowledge the technical assistance of Miss R. Kellerhals, J. Eisele, I. Looser and K. Naumann. This work has been supported in part (W.P.) by the Swiss National Fund for Scientific Research, Grant No. 3.8750.72.

### REFERENCES

Abraham, G. E. (1969) Solid-phase Radioimmunoassay of Estradiol-17. *J. clin. Endocrin.*, **29**, 866.

Abraham, G. E. (1974) Radioimmunoassay of Steroids in Biological Materials. *Acta endocrin.* (*Kbh*), Suppl., **183**, 1.

Barraclough, C. A. (1961*a*) Production of Anovulatory Sterile Rats by Single Injection of Testosterone Propionate. *Endocrinology*, **68**, 62.

Barraclough, C. A. (1961*b*) Evidence that the Hypothalamus is Responsible for Androgen-induced Sterility in the Female Rat. *Endocrinology*, **68**, 68.

Barraclough, C. A. & Leathem, J. H. (1954) Infertility Induced in Mice by a Single Injection of Testosterone Propionate. *Proc. Soc. exp. Biol. Med.*, **85**, 673.

Buus, O. (1968) Double Isotope Technique for Determination of the Individual Corticosteroids in Human Plasma. *Excerpta med.*, **157**, 107.

Dale, E. & Scutchfield, F. D. (1968) Adrenal Lipid and Plasma Corticosterone Depletion after 7,12-dimethylbenz(a)anthracene Administration to the Albino Rat. *Experientia*, **24**, 723.

Dunn, T. B. (1954) Normal and Pathologic Anatomy of the Reticular Tissue in Laboratory Mice, with a Classification and Discussion of Neoplasms. *J. natn. Cancer Inst.*, **14**, 1281.

Gardner, W. U. (1953) Hormonal Aspects of Experimental Tumorigenesis. *Adv. Cancer Res.*, **1**, 173.

Haran-Ghera, N. (1973) Relationship between Tumor Cell and Host in Chemical Leukemogenesis. *Nature, New Biol.*, **246**, 84.

Haran-Ghera, N., Kotler, M. & Meshorer, A. (1967) Studies on Leukemia Development in the SJL/J Strain of Mice. *J. natn. Cancer Inst.*, **34**, 653.

Kaplan, H. S. (1950) Influence of Thymectomy, Splenectomy and Gonadectomy on Incidence of Radiation-induced Lymphoid Tumors in Strain C57BL Mice. *J. natn. Cancer Inst.*, **11**, 83.

Kirschbaum, A. (1951) Rodent Leukemia: Recent Biological Studies. *Cancer Res.*, **11**, 741.

Krarup, T. (1970) Effect of 9 : 10-dimethyl-1 : 2 benzanthracene on the Mouse Ovary. *Acta endocr.*, **64**, 489.

Lacassagne, A. (1937) Sarcomas Lymphoides Apparus chez des Souris Longuement Traitées par des Hormones Oestrogènes. *C. r. Séanc. Soc. Biol.*, **126**, 193.

Law, L. W. & Miller, J. H. (1950) The Influence of Thymectomy on the Incidence of Carcinogen-induced Leukemia in Strain DBA Mice. *J. natn. Cancer Inst.*, **11**, 425.

Martinez, C. (1964) Effect of Early Thymectomy on Development of Mammary Tumours in Mice. *Nature, Lond.*, **203**, 1188.

Mittra, I. & Hayward, J. L. (1974*a*) Hypothalamic–pituitary–thyroid Axis in Breast Cancer. *Lancet*, i, 885.

Mittra, I. & Hayward, J. L. (1974*b*) Hypothalamic–Pituitary–Prolactin Axis in Breast Cancer. *Lancet*, i, 889.

Mühlbock, O. & Boot, L. M. (1958) The Mechanism of Hormonal Carcinogenesis. In *Ciba Foundation Symposium on Carcinogenesis. Mechanisms of action.* London: Churchill, p. 83.

Murphy, E. D. (1963) SJL/J, a New Inbred Strain of Mouse with a High, Early Incidence of Reticulum-cell Neoplasms. *Proc. Am. Ass. Cancer Res.*, **4**, 46.

Murphy, B. E. P. & Jachan, C. (1965) The Determination of Thyroxine by Competitive Protein-binding Analysis Employing an Anion Exchange Resin and Radiothyroxine. *J. Lab. clin. Med.*, **66**, 161.

Murphy, B. E. P., Pattee, C. J. & Gold, A. (1966) Clinical Evaluation of a New Method for the Determination of Serum Thyroxine. *J. clin. Endocrin.*, **26**, 247.

Papaioannou, A. N. (1974) *The Etiology of Human Breast Cancer.* Berlin: Springer Verlag.

Pierpaoli, W. & Besedovsky, H. O. (1975) Role of the Thymus in Programming of Neuroendocrine Functions. *Clin. exp. Immunol.*, **20**, 323.

Pierpaoli, W., Haran-Ghera, N., Bianchi, E., Müller, J., Meshorer, A. & Bree, M. (1974) Endocrine Disorders as a Contributory Factor to Neoplasia in SJL/J Mice. *J. natn. Cancer Inst.*, **53**, 731.

Pierpaoli, W. & Haran-Ghera, N. (1975) Prevention of Induced Leukemia in Mice by Immunological Inhibition of Adenohypophysis. *Nature, Lond.*, **254**, 334.

Pierpaoli, W., Kopp, H. G. & Bianchi, E. (1976) Interdependence of Thymic and Neuroendocrine Functions in Ontogeny. *Clin. exp. Immunol.*, **24**, 501.

Pierpaoli, W. & Sorkin, E. (1972*a*) Inhibition of Growth of Methylcholanthrene-induced Mammary Carcinoma in Rats by Anti-adenohypophysis Serum. *Nature, New Biol*, **238**, 58.

Pierpaoli, W. & Sorkin, E. (1972*b*) Alteration of Adrenal Cortex and Thyroid in Mice with Congenital Absence of the Thymus. *Nature, New Biol.*, **238**, 282.

Silberberg, M., Silberberg, R. & Leidler, H. V. (1951) Effects of Anterior Hypophyseal Transplants on Intrasplenic Ovarian Grafts. *Cancer Res.*, **11**, 624.

Sinha, Y. N., Selby, F. W. & Vanderlaan, W. P. (1974) The Natural History of Prolactin and GH Secretion in Mice with High and Low Incidence of Mammary Tumors. *Endocrinology*, **94**, 757.